# Adherence to PKU guidelines among patients with phenylketonuria: A cross-sectional national multicenter survey-based study in Argentina, Brazil, and Mexico

**DOI:** 10.1016/j.ymgmr.2023.101026

**Published:** 2023-11-21

**Authors:** Ana Chiesa, Norma Spécola, Monique Poubel, Marcela Vela-Amieva, Elaina Jurecki, Daniel RF Vilela, Débora Mesojedovas, Giovanna Cavalcanti Carneiro, Hernán Eiroa, Keila Hayashi Nakamura, Marcela Lopes de Almeida, Roberta Brandão Cunha, Tatiana Amorim, Ida Vanessa Doederlein Schwartz

**Affiliations:** a Centro de Investigaciones Endocrinológicas Dr Cesar Bergadá CEDIE Fundación de Endocrinología Infantil/ Hospital de Niños R Gutierrez, Argentina; b Unidad de Metabolismo, Hospital de Niños Sor Ludovica de La Plata, Argentina; c Hospital de Apoio de Brasília, Brazil; d Laboratorio de Errores Innatos del Metabolismo y Tamiz del Instituto Nacional de Pediatría, Mexico; e Genetic Pharmaceutical Consulting, USA; f BioMarin Farmacêutica do Brasil, LTDA, Brazil; g Formerly at BioMarin Farmacêutica do Brasil, LTDA, Brazil; h Hospital Coronel Pedro Germano, Argentina; i Servicio de Errores Congénitos del Metabolismo, Hospital de Pediatria Dr Juan P. Garrahan, Argentina; j Reference Service in Newborn Screening of Estadual University of Campinas UNICAMP, Brazil; k Hospital das Clínicas de Ribeirão Preto, Faculdade de Medicina da Unversidade de São PauloRibeirão Preto, Brazil; l Associação de Pais e Amigos dos Excepcionais Rio de Janeiro, Rio de Janeiro, Brazil; m Associação de Pais e Amigos dos Excepcionais Salvador, Brazil; n Department of Genetics, Universidade Federal do Rio Grande do Sul, Brazil

**Keywords:** Adherence, PKU, Latin America, Blood Phe, Clinic visits, Blood testing frequency

## Abstract

**Objective:**

To characterize adherence to Phenylketonuria (PKU) management practices among PKU patients treated at reference sites around Argentina, Brazil, and Mexico.

**Methods:**

This is a retrospective, observational, multicenter, and multinational survey-based study using aggregate data. From an initial list of 40 sites, 22 clinicians expressed interest in completing the survey, with 20 clinicians from 20 unique sites fulfilling all the study criteria. The Survey contained 28 questions, including respondent's clinic characteristics, clinic PKU treatment recommendations, and patient adherence to clinic recommendations. Survey was available in local languages, and the respondents were asked to consult their clinic records to complete their responses. Adherence was assessed by target blood phenylalanine (Phe), target blood testing frequency, and clinic visits.

**Results:**

A total of 1077 (out of 1377) actively managed PKU patients (seen in the clinic in the last 3 years) from 13 clinics in Brazil, six in Argentina, and one in Mexico were analyzed. Upper blood Phe target was set over 360 μMol/L in 70% of the clinics for adult patients. Around 40% of the patients >30 years old had Phe blood tests done twice a year or less, with 60% of the clinics recommending semestral visits for adults <30 years old. Twice a month was the most common frequency of visits for <1 year old. The COVID-19 pandemic was a disruptor for frequency of visits and exams.

**Conclusions:**

These results show that there is still room for improvement in terms of adherence, namely in adults and older children. More efforts must be made to educate patients and healthcare professionals about the importance of treatment adherence, accompanied by public policies that expand access to pharmacological and dietary treatment with diversity and quality to improve adherence to adequate blood Phe levels.

## 1 Introduction

Phenylketonuria (PKU) is a rare, debilitating, and inherited metabolic disorder caused by a deficiency of the phenylalanine (Phe) hydroxylase enzyme (PAH), of which tetrahydrobiopterin (BH4) is a cofactor, that is responsible for the conversion of Phe to tyrosine (Tyr). [1] Phe is an essential amino acid obtained solely through diet or proteolysis, and is indispensable for protein synthesis as well as for the synthesis of Tyr and its secondary derivatives, including dopamine, norepinephrine, and melanin. [2] Synthesis of Tyr, through hydroxylation of Phe by PAH takes place predominantly in the liver. Thus, PAH deficiency leads to increased concentration of Phe in the blood and brain. [3,4]

PKU is considered one of the most common inherited metabolic diseases in some regions of Europe, with a mean prevalence of 1:10,000 newborns, while in Latin America (LatAm), it varies from about 1:15,000 to 1:50,000 births. [[5], [6], [7], [8]]

The consequences of untreated PKU include intellectual disability, microcephaly, motor deficits, autism, seizures, developmental problems, aberrant behavior, and psychiatric symptoms. [7] Therefore, newborn screening programs (NBS) for early diagnosis and disease management are essential to reduce the impact of elevated Phe. Brazil initiated their NBS program in 1976 and nationwide mandatory screening was implemented in the 1990s, with national coverage established by 2001, leading to a significant increase in newborn screening of the general population to 85.8%. [9] In Argentina, regional screening began in 1995, achieving national coverage by 2006, resulting in 90% of newborns screened. [10] For Mexico, the availability and implementation of NBS vary by region with coverage provided individually from each of the six healthcare systems, and lacks national coordination. Nevertheless, coverage currently reaches >70% of newborns. [10] Overall, 14 out of the 20 countries in LatAm have implemented a NBS program for PKU.

The cornerstone of disease management is a Phe-restricted diet supplemented with medical foods fortified with Phe-free amino acids [1]. Dietary treatment varies according to the severity of PKU and may now include pharmacological treatment, since successful long-term dietary management is a challenge. Most patients follow a diet that is very low in natural proteins with limited to no animal protein sources [7]. Pharmacotherapy has been shown to improve outcomes in individuals with PKU. Some PKU patients have been shown to be responsive to sapropterin (Kuvan®, BioMarin Pharmaceutical Inc., Novato, CA, USA) a synthetic form of tetrahydrobiopterin (BH4 cofactor therapy), which stimulates residual PAH activity and allows treatment responders to have an increase in dietary protein intake. Even so, sustained adherence is challenging given the extremely restrictive nature of the diet in those that are not responsive or minimally responsive to BH4. [7,11]. An enzyme substitution therapy, pegvaliase (Palynziq™, BioMarin Pharmaceutical Inc., Novato, CA, USA), has also been shown to effectively lower blood Phe over 16 years old and in adults with PKU. The importance of pharmacotherapies in supporting outcomes in patients with PKU, such as quality of life (QoL), was recently addressed in a systematic review [12]. Currently, sapropterin and pegvaliase [13], are not widely available across the region (e.g., sapropterin is available in Argentina, Brazil, and Mexico, but pegvaliase, is not).

Clinical recommendations and treatment guidelines are available for most regions. However, LatAm lacks guidelines across countries. There is a lack of consensus on some key issues, such as target blood Phe concentrations, and frequency of blood testing frequency and annual clinical appointments. Patient journey varies between countries in LatAm due to significant differences in the organization of the national healthcare systems, impacting access to care. [3,14] A study in the US was conducted to evaluate adherence to clinical guidelines for the management of patients with PKU. [15] Results identified the need to increase adherence to guidelines in order to achieve optimal metabolic control and patient outcomes. Increased patient education and training of medical staff are key factors along with the need to increase the frequency of follow-up in patients who are not actively managed at reference centers.

The evidence concerning the need for adoption and adherence to international guidelines for the treatment of PKU is limited for LatAm. A 2012 epidemiology study provided much-needed data on the prevalence and incidence of PKU in 20 countries in the region. [16] More recently, landmark publications [9,10,12,17,18] provided updates on unmet needs in the region, along with the challenges that many individuals with PKU still face. These include the need to improve the range of therapeutic options available, access to blood Phe testing, healthcare providers knowledgeable in PKU management, greater availability of resources, and the availability of psychiatrists/psychologists in reference centers.

This study aimed to address the adherence to PKU management practices among PKU patients treated at reference sites located in Argentina, Brazil, and Mexico. These countries were selected based on the status of PKU NBS in these countries as compared to that in other LatAm countries, resulting in higher numbers of diagnosed and treated patients, as well as in the PKU and total population size. Adherence to PKU management among individuals with PKU was assessed through a survey to clinicians (physicians and dietitians) who are specialized in the treatment and management of PKU. Adherence to PKU management practices was assessed using three PKU outcomes as indicators: 1) target blood Phe concentrations, 2) the frequency of blood Phe concentration testing, and 3) the frequency of clinical consultations. Moreover, this study explored adherence to site management practices for specific populations, including age cohorts -from newborns to adults- and pregnant patients with PKU. Understanding the differences and similarities of treatment and follow-up protocols for patients with PKU in different LatAm countries and mapping treatment adherence is important to correlate with clinical outcomes throughout the life cycle. However, this study does not aim to provide a characterization of the LatAm region, as this was performed in a recent study. [17] Lastly, results of this survey will help to inform health authorities on the need to develop LatAm PKU treatment guidelines and increase access to better care.

## 2 Materials and methods

### 2.1 Study design and participants

This multinational, regional, survey-based cross-sectional study with retrospective data collection was designed to characterize patient adherence to PKU management practices in three LatAm countries (Argentina, Brazil, and Mexico) as these countries have the most developed PKU diagnostic and management programs. The option for these three countries followed criteria such as population size, proportion of PKU patients, professional experience, maturity and development of the NBS program and years of expertise in clinical treatment of PKU. Data were collected through an online survey targeting clinicians (physicians or dietitians) from PKU reference sites. In addition to a willingness to participate in the survey, clinicians were required to meet the following inclusion criteria: affiliation with a site that had at least 5 actively managed PKU patients (actively managed defined as patients that have been followed at the clinic within the past 3 years), managing PKU patients for at least three years, and being able to access patient records. Clinicians were excluded if they did not consider themselves a specialist in PKU treatment. Only one responder (physician/dietitian) was allowed per site.

### 2.2 Survey

This survey was conducted from April through October 2021 and included twenty questions in addition to eight screening questions to ensure that they met the eligibility criteria*.* Data collected included the respondent's clinic characteristics (country/state, number of PKU patients, number and norm of full-time staff that treat PKU, years of experience with PKU treatment), PKU treatment recommendations (target blood Phe, target blood testing frequency, target clinic visit frequency), and patient adherence to clinic recommendations. Treatment recommendations and adherence questions were broken down by specific patient age groups (ages <1, 1–4, 5–12, 13–15, 16–17, 18–29, 30+ years) and for patients who were pregnant or planning on becoming pregnant within 12 months. Respondents were asked to define patient adherence to target blood Phe recommendations based on the average Phe concentrations obtained over the past year or the most recent blood Phe level obtained in the past 3 years (the latter due to the global pandemic starting in 2020 and the timing of the survey in 2021). Respondents were encouraged to refer to their clinic's patient database, patient medical charts, and clinic staff to provide accurate information. The full survey can be accessed online (Supplementary Survey Text). The survey was available to clinicians in Portuguese, Spanish, and English*.* An exemption from local Independent Review Board approval was granted since (I) the survey did not request individualized patient outcome data, (II) the answers provided by the clinicians were anonymized, and (III) the findings disclosed in this manuscript resulted from aggregated data.

### 2.3 Study recruitment

The questionnaire was sent to a total of 40 clinicians (physicians and dietitians) from PKU clinics, with robust NBS programs and consequently a high incidence of PKU diagnosis - as identified by previous research. In addition, these sites were selected due to the high number of managed PKU patients besides being reference centers in each region. Respondents from 22 unique clinics replied to the survey invitation (55% response rate) and completed the survey. Two of these did not meet the inclusion criteria defined in the protocol for the minimum number of patients and, therefore, were excluded from the analysis, leaving a total of 20 participating sites: 13 in Brazil, 6 in Argentina, and one in Mexico.

### 2.4 Statistical analysis

Quantitative variables were summarized as mean, median, standard deviation, minimum and maximum, and qualitative variables were summarized as absolute frequency and percentage, overall and by subgroups. Age-based subgroups included a) < 1 year of age; b) between 1 and 4 years of age; c) between 5 and 12 years of age; d) between 13 and 15 years of age; e) between 16 and 17 years of age; f) between 18 and 29 years of age; g) ≥ 30 years of age. An additional subgroup was formed of patients who were pregnant/planning to become pregnant within 12 months. The patients included in the pregnant/ planning on becoming pregnant group were also included in the appropriate age-based group. For some analyses related to adherence, an aggregate group of ages between 13 and 17 years old was created. This group represents the core ages of adolescence, and the number of cases makes it more representative for statistical analysis.

The target range for non-adherent patients considered for this analysis was based on the American College of Medical Genetics (ACMG) guidelines [3] used as the standard value for the global analysis. Other reference values, such as the upper target blood Phe level of 600 μMol/L suggested in European Guidelines [7], were also considered, though in this evaluation a patient was considered non-adherent (above target range) if the blood Phe level was above 360 μMol/L. However, it must be mentioned that in the case of Argentina, the reference value most used (600 μMol/L) is less restrictive, for adult non-pregnant patients. Therefore, this should be considered when evaluating the overall results depicted in this study given the higher blood Phe target in Argentina. Country-level data presents both thresholds, and for Argentina, the upper limit of 600 μMol/L should be considered, although one center uses 360 μMol/L as the upper limit. According to the Brazilian Ministry of Health for PKU, the Phe upper limit target levels are 600 μMol/L for patients above 12 years old, while 0–12 years old and pregnant patients have an upper target of 360 μMol/L.

There was no imputation of missing data. Statistical significance was set at 5%. Statistical analysis was performed using SAS® (version 9.4, SAS Institute Inc., Cary).

## 3 Results

### 3.1 Site/ institution's general characteristics

Most of the 20 sites were in Brazil (13/20, 65%), representing the country's geographic regions. Five sites were in the northeast region of Brazil, four in the southeast region, two in the midwest (*n* = 2, 15.4%), and the remaining two were in the north and south regions, respectively. Six sites were in Argentina, three in Buenos Aires, and the remaining three were in central Argentina, the Patagonia region, and Cuyo. In Mexico, the only participating site was in the central region of the country (Mexico City).

### 3.2 Respondent clinician characteristics

Across the three countries, half of the responding clinicians were specialized in pediatrics, including pediatricians (*n* = 4), pediatric endocrinologists (n = 4), and pediatric neurologists (n = 2). In Brazil, almost half of the respondents (*n* = 6, 46.2%) were dietitians, followed by geneticists (*n* = 3, 23.1%), pediatric (n = 3, 23.1%) endocrinologists (n = 3, 23.1% each) and a pediatric neurologist (*n* = 1, 7.7%). In Argentina, almost two-thirds were pediatricians (n = 4, 66.7%), and the remaining two were one pediatric endocrinologist (n = 1, 16.7%) and one pediatric neurologist (n = 1, 16.7%). In Mexico, the respondent clinician was a metabolism physician. All clinicians were specialized in the treatment of patients with PKU and reported managing patients with PKU for a mean time of 12 years (SD: 9.8 years). The respondent clinicians' average experience in treating PKU was 25.00 years in Mexico (n = 1), 19.0 (SD: 12.0, range: 4.0 to 35.0) years in Argentina, and 8.15 (SD: 6.0, range: 3.0 to 20.0) years in Brazil.

### 3.3 Number of managed PKU patients

Clinicians across the LatAm region reported actively managing 1077 PKU patients, in which 27% of the patients were adults (Table 1). The total number of all patients ever followed by the clinics was 1317 (28% adults), including those not seen in the last 3 years. Approximately 0.7% of the actively managed patients (and 2.7% of adults) were described as pregnant or planning to become pregnant in the upcoming 12 months. In Brazil, 634 patients with PKU were actively followed among all 13 Brazilian sites. The age group with the highest number of patients was between 5 and 12 years old with 28.9% (*n* = 183), and patients 30 years of age or older represented just under 10% (*n* = 59, 9.3%) of those actively managed. Only 1.1% (*n* = 7) were pregnant or planning on becoming pregnant within 12 months. In Argentina, 383 patients with PKU were actively managed at the 6 participating clinics. Over one-quarter of these patients were between 13 and 17 years of age (*N* = 109, 28.5%), followed by 18 to 29 years (*n* = 95, 24.8%). The youngest and older patient age groups were the least represented; 4.4% (*n* = 17) of the patients were <1 year of age, and 3.7% (*n* = 14) were 30 years old or more. In Mexico, the participating site actively followed 60 patients diagnosed with PKU. Approximately 77% of the patients were between 1 and 12 years old (*n* = 46, 76.7%), only 5.0% (*n* = 3) of those actively managed were between 18 and 29 years of age, and the site reported no actively managed patients above the age of 30 years.

**Table 1:** Site/ Institution's total number of managed PKU patients.

	Brazil (*N* = 767)	Argentina (*N* = 430)	Mexico (*N* = 120)	Total (*N* = 1317)
**No. of patients diagnosed with PKU in the clinic and actively managed**	634	383	60	1077
**Patients diagnosed with PKU in the clinic actively managed by age group, n (%)**				
<1 year of age	18 (2.8%)	17 (4.4%)	4 (6.7%)	39 (3.6%)
1 to 4 years of age	104 (16.4%)	60 (15.7%)	22 (36.7%)	186 (17.3%)
5 to 12 years of age	183 (28.9%)	88 (23.0%)	24 (40.0%)	295 (27.4%)
13 to 17 years of age	149 (23.5%)	109 (28.5%)	7 (11.7%)	265 (24.6%)
18 to 29 years of age	121 (19.1%)	95 (24.8%)	3 (5.0%)	219 (20.3%)
≥30 years of age	59 (9.3%)	14 (3.7%)	0 (0.0%)	73 (6.8%)
**Patients diagnosed with PKU in the clinic and actively managed and pregnant/ planning on becoming pregnant soon, n (%)**				
Yes	7 (1.1%)	1 (0.3%)	0 (0.0%)	8 (0.7%)
No	627 (98.9%)	382 (99.7%)	60 (100.0%)	1069 (99.3%)
Total	634	383	60	1077
**Patients diagnosed with PKU in the clinic in total (including those that are actively followed and inactively followed) by age group, n (%)**				
<1 year of age	29 (3.8%)	17 (4.0%)	6 (5.0%)	52 (3.9%)
1 to 4 years of age	142 (18.5%)	61 (14.2%)	28 (23.3%)	231 (17.5%)
5 to 12 years of age	204 (26.6%)	97 (22.6%)	30 (25.0%)	331 (25.1%)
13 to 17 years of age	180 (23.5%)	117 (27.2%)	35 (29.2%)	332 (25.2%)
18 to 29 years of age	128 (16.7%)	114 (26.5%)	11 (9.2%)	253 (19.2%)
≥30 years of age	84 (11.0%)	24 (5.6%)	10 (8.3%)	118 (9.0%)
Total	767	430	120	1317
**Patients diagnosed with PKU in the clinic not actively managed by age group, n (%)**				
<1 year of age	11 (8.3%)	0 (0.0%)	2 (3.3%)	13 (5.4%)
1 to 4 years of age	38 (28.6%)	1 (2.1%)	6 (10.0%)	45 (18.8%)
5 to 12 years of age	21 (15.8%)	9 (19.1%)	6 (10.0%)	36 (15.0%)
13 to 17 years of age	31 (23.3%)	8 (17.0%)	28 (46.7%)	67 (27.9%)
18 to 29 years of age	7 (5.3%)	19 (40.4%)	8 (13.3%)	34 (14.2%)
≥30 years of age	25 (18.8%)	10 (21.3%)	10 (16.7%)	45 (18.8%)
Total	133	47	60	240

PKU – Phenylketonuria.

n represents the number of clinics contributing to the summary. Missing values are not considered in the percentage's calculation.

### 3.4 PKU clinic staffing

The staffing characteristics and specialties of the PKU reference sites overall and for each participating country are shown in Table 2. A total of 109 clinicians, with a mean of 5.45 full-time healthcare professionals (SD = 3.15, range: 0 to 14) were reported per site. In Brazil, there were an average of 5.77 healthcare professionals (SD = 3.79, range: 0 to 14) per site, while in Argentina, 4.50 healthcare professionals (SD = 1.22, range: 3 to 6) per site; and seven in the single site in Mexico.

**Table 2:** PKU clinic staffing.

	Brazil (*N* = 75)	Argentina (*N* = 27)	Mexico (N = 7)	Total (N = 109)
**No. of the following specialties that treat PKU patients and work full-time in the clinics, by specialty n (%) and - average per site**				
Physicians	23 (30.7%) – 1.77	14 (51.9%) – 2.33	3 (42.9%) – 3.00	40 (36.7%) – 2.00
Geneticists ^a)^	10 (43.5%) – 0.77	–	–	10 (43.5%) – 0.77
Dietitians	14 (18.7%) – 1.08	7 (25.9%) – 1.17	2 (28.6%) – 2.00	23 (21.1%) – 1.15
Social workers	13 (17.3%) – 1.00	4 (14.8%) – 0.67	0 (0.0%) – 0.00	17 (15.6%) – 0.85
Psychologists / neuropsychologists	10 (13.3%) – 0.77	1 (3.7%) – 0.17	0 (0.0%) – 0.00	11 (10.1%) – 0.55
Nurse practitioners	15 (20.0%) - 1.15	1 (3.7%) – 0.17	0 (0.0%) – 0.00	16 (14.7%) – 0.80
Genetic counselors ^b)^	–	0 (0.0%) – 0.00	2 (28.6%) – 2.00	2 (1.8%) – 0.29
Total	75	27	7	109

PKU – Phenylketonuria. n represents the number of clinics contributing to the summary. Missing values are not considered in the percentage's calculation. Q1: quartile 1. Q3: quartile 3.

a) In Brazil, the number of full-time geneticists was collected instead of genetic counselors. The number of physicians also include geneticists.

b) The number of full-time genetic counselors was only collected in Argentina and Mexico.

Dietitians, who are at the forefront of the management of PKU, appear to be available in similar proportions in Brazil and Argentina. At the same time, Mexico presented a higher availability of this specialty.

### 3.5 Current PKU management practices

#### 3.5.1 Blood Phe: clinic recommendations and patient adherence

Survey respondents were asked about the lower and upper limits of the recommended target range for blood Phe for each group. (Table 3) Age groups between <1 year to 16 to 17 years and in the group of pregnant women, showed a consensus on the lower limit for the target concentration of blood Phe, with approximately 95% of clinics recommending 120 μMol/L, which is aligned with ACMG guidelines. This percentage reduced to approximately 89% in the 18–29 and 30+ age groups. The recommended upper target was reported to be more variable. While all sites recommended a target of 360 μMol/L or less for patients under 4 years of age and for the pregnancy group, the upper- recommended target increased for older age groups. For patients aged 13 to 17 years old, nearly half of the sites recommended an upper target over 360 μMol/L and approximately 70% (*n* = 13) of sites have higher targets for adult patients. Among all participating clinics, 5.0% (n = 1) did not have a target blood Phe level for patients between 18 and 29 years, and 10% (*n* = 2) reported not having this target defined for patients who were 30 years or older nor for patients that were pregnant or planning on becoming pregnant within the next 12 months.

**Table 3:** Current PKU management practices used by PKU reference sites.

	< 1 year (*N* = 20)	1 to 4 years (*N* = 20)	5 to 12 years (N = 20)	13 to 15 years (N = 20)	16 to 17 years (N = 20)	18 to 29 years (N = 20)	≥ 30 years (N = 20)	Pregnant/ planning on becoming pregnant soon (N = 20)
**Current recommended blood Phe target range for PKU patients, n (%)**								
**Lower Target**								
≤120 μMol/L	20 (100%)	20 (100%)	19 (95.0%)	19 (95.0%)	19 (95.0%)	17 (89.5%)	16 (88.9%)	17 (94.4%)
>120 μMol/L	0 (0.0%)	0 (0.0%)	1 (5.0%)	1 (5.0%)	1 (5.0%)	2 (10.5%)	2 (11.1%)	1 (5.6%)
Total	20	20	20	20	20	19	18	18
**Upper Target**								
≤360 μMol/L	20 (100%)	20 (100%)	17 (85.0%)	10 (50.0%)	9 (45.0%)	6 (31.6%)	5 (27.8%)	16 (88.9%)
361–599 μMol/L	0 (0.0%)	0 (0.0%)	0 (0.0%)	2 (10.0%)	2 (10.0%)	1 (5.3%)	1 (5.6%)	0 (0.0%)
≥600 μMol/L	0 (0.0%)	0 (0.0%)	3 (15.0%)	8 (40.0%)	9 (45.0%)	12 (63.2%)	12 (66.7%)	2 (11.1%)
Total	20	20	20	20	20	19	18	18
**Clinic does not have a target Phe goal**^**a)**^	0 (0.0%)	0 (0.0%)	0 (0.0%)	0 (0.0%)	0 (0.0%)	1 (5.0%)	2 (10.0%)	2 (10.0%)
**Recommended frequency of blood Phe tests per year to assess Phe levels for PKU, n (%)**^**b)**^								
Weekly or more often	6 (31.6%)	3 (15.8%)	0 (0.0%)	0 (0.0%)	0 (0.0%)	0 (0.0%)	0 (0.0%)	5 (29.4%)
Twice a month	7 (36.8%)	4 (21.1%)	1 (5.3%)	2 (10.5%)	0 (0.0%)	0 (0.0%)	0 (0.0%)	2 (11.8%)
Monthly	5 (26.3%)	7 (36.8%)	7 (36.8%)	4 (21.1%)	6 (31.6%)	4 (21.1%)	3 (16.7%)	7 (41.2%)
Trimonthly (every 3 months)	1 (5.3%)	5 (26.3%)	11 (57.9%)	12 (63.2%)	11 (57.9%)	12 (63.2%)	11 (61.1%)	3 (17.6%)
Twice a year	0 (0.0%)	0 (0.0%)	0 (0.0%)	1 (5.3%)	2 (10.5%)	3 (15.8%)	3 (16.7%)	0 (0.0%)
Annually	0 (0.0%)	0 (0.0%)	0 (0.0%)	0 (0.0%)	0 (0.0%)	0 (0.0%)	1 (5.6%)	0 (0.0%)
Greater than annually	0 (0.0%)	0 (0.0%)	0 (0.0%)	0 (0.0%)	0 (0.0%)	0 (0.0%)	0 (0.0%)	0 (0.0%)
Total	19	19	19	19	19	19	18	17
Missing values	0	0	0	0	0	0	1	2
**Recommended number of clinic visits per year for PKU patients, n (%)**^**c)**^								
Weekly or more often	1 (5.3%)	0 (0.0%)	0 (0.0%)	0 (0.0%)	0 (0.0%)	0 (0.0%)	0 (0.0%)	1 (5.9%)
Twice a month	8 (42.1%)	5 (26.3%)	1 (5.3%)	1 (5.3%)	0 (0.0%)	0 (0.0%)	0 (0.0%)	7 (41.2%)
Monthly	6 (31.6%)	3 (15.8%)	0 (0.0%)	0 (0.0%)	1 (5.3%)	1 (5.3%)	1 (5.9%)	2 (11.8%)
Trimonthly (every 3 months)	3 (15.8%)	9 (47.4%)	10 (52.6%)	10 (52.6%)	10 (52.6%)	7 (36.8%)	5 (29.4%)	6 (35.3%)
Twice a year	1 (5.3%)	2 (10.5%)	8 (42.1%)	8 (42.1%)	8 (42.1%)	11 (57.9%)	11 (64.7%)	1 (5.9%)
Annually	0 (0.0%)	0 (0.0%)	0 (0.0%)	0 (0.0%)	0 (0.0%)	0 (0.0%)	0 (0.0%)	0 (0.0%)

a) The percentages were calculated considering the total number of clinics (N).

b) Only clinics that have a recommended frequency of blood tests are included. Includes blood tests obtained on clinic days and blood tests obtained between appointments.

c) Only clinics that have a recommended number of clinic visits per year for PKU patients are included.

PKU – Phenylketonuria. Phe – phenylalanine.

N represents the number of clinics contributing to the summary. Missing values are not considered in the percentage's calculation.

**Fig. 1:**
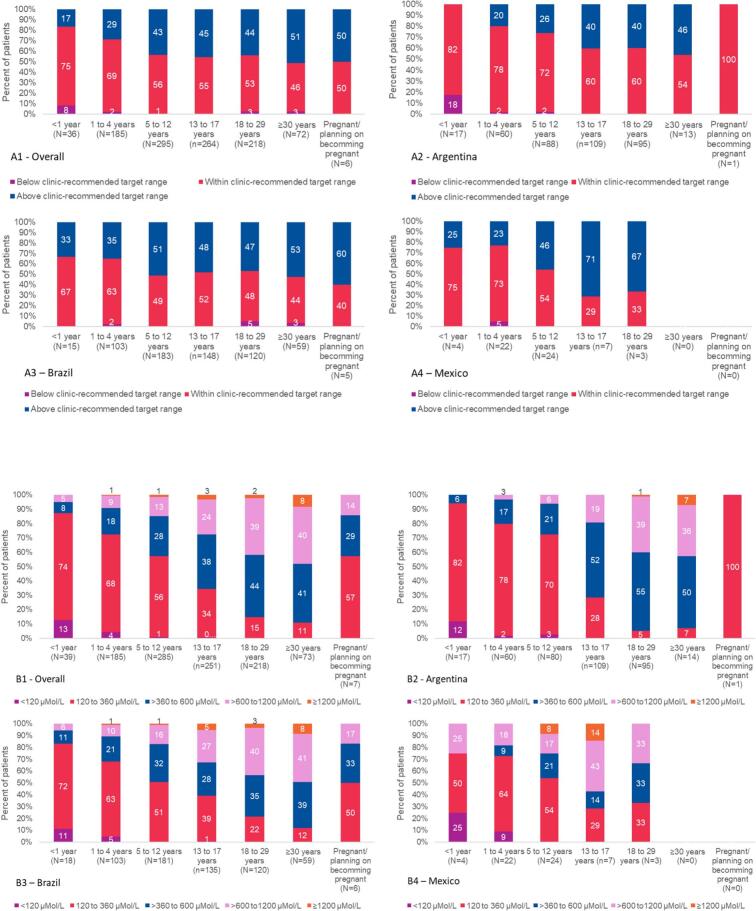
Patient distribution concerning recommended blood Phe concentrations obtained over the previous year (below, within or above clinic-recommended target), by age and pregnancy status – Overall and by country. A. Patient adherence to clinic-recommended blood Phe target concentrations – Overall and by country. B. Patient distribution according to actual blood Phe concentrations.

The adherence to the target Phe range was assessed based on the PKU management practices for each site, represented in Fig. 1a, and based on pre-specified guideline target levels, represented in Fig. 1b. Overall, the percentage of patients above the target Phe range defined by each clinic increased with age. For patients 30 years or older (*n* = 37, 51.4%) and in the small group of pregnant patients (n = 3, 50.0%), around half of them were above the blood Phe target. Considering only the PKU patients that are actively managed, >80% of the patients 16 years or older had an average blood Phe level above 360 μMol/L. This percentage was 54.4% for patients between 13 and 15, approximately 43% for children 5 to 12 years old and 43% for pregnant patients, and <30% for younger children (<5 years of age).

The percent of patients above the recommended blood Phe levels stratified by the upper limit of clinic recommendations for the age groups from 18 to 29 years old and 30 years old or more is shown in Fig. 1. The proportion of patients above target for clinics targeting 360 μMol/L in patients between 18 and 29 years is around 65% and almost 80% for patients 30 years or older. This proportion above the target range decreases to close to 50% for both age groups in clinics targeting 600 μMol/L.

A specific note should be made for Argentina. As shown in Fig. 1. B2, if considering the less conservative target of 600 μMol/L, the proportion of adult patients not adherent to recommendations decreases significantly, and in some of the age cohorts (i.e., < 1 year old), no patients can be considered non-adherent. In some of the older age cohorts, the results show a much lower proportion of non-adherence. For Brazil (Fig. 1. B3), the overall proportion of adherence is similar to the country-level data. As for Mexico (Fig. 1. B4), no patients over 30 years old and pregnant/intending to become pregnant were included, and only four patients were under 1 year old.

#### 3.5.2 Blood Phe testing frequency: clinic recommendations and patient adherence

**Fig. 2:**
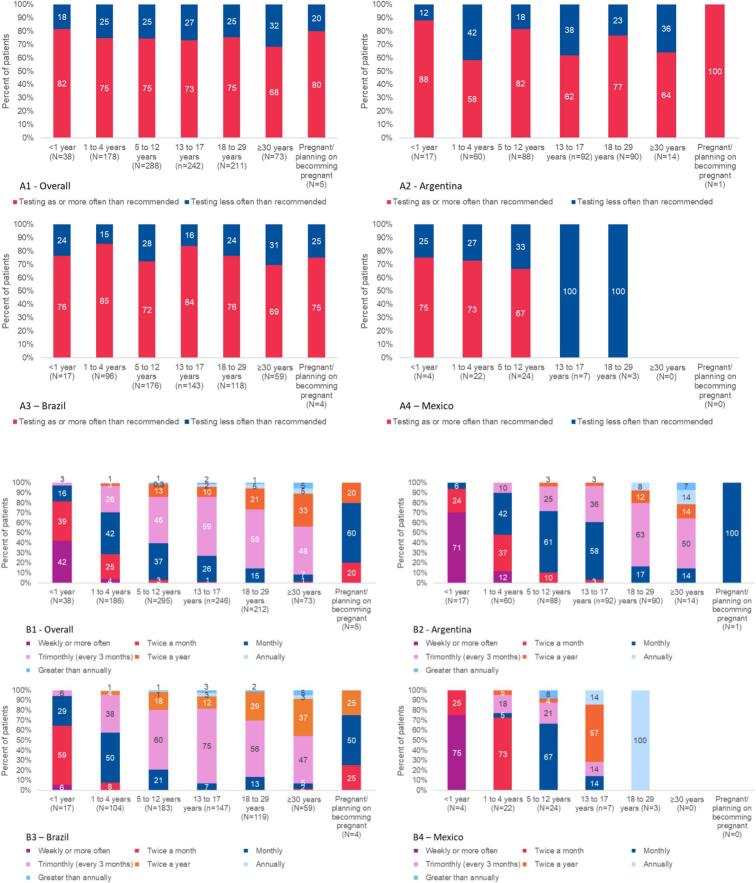
Patient distribution according to actual blood Phe testing frequency, by age and pregnancy status – Overall and by country. A. Patient adherence to blood Phe testing frequency recommended by their clinic. B. Patient distribution according to actual blood Phe testing frequency.

**Fig. 3:**
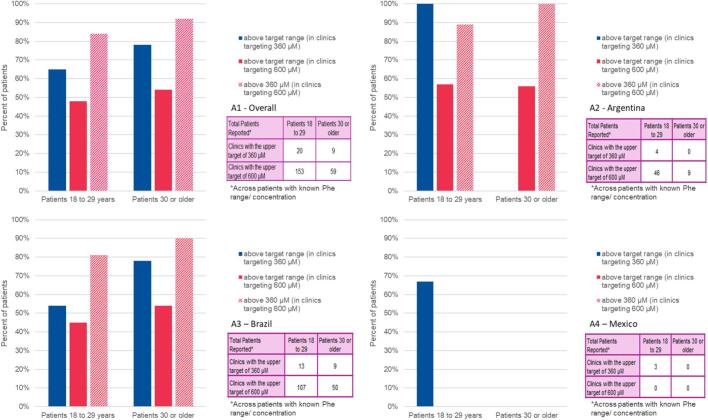
Relationship between clinic target blood Phe concentration and patient adherence – Overall and by country.

Ninety-five percent of clinics that participated in the survey (19/20) had a protocol in place for recommended frequency of blood Phe testing for patients under the age of 29; this fell to 90% for those above the age of 30 years and to 85% for the pregnancy group (Table 3). Overall, the most common frequency of testing for each age group (Fig. 2b) was concordant with the most commonly recommended (Fig. 2a) frequency of testing: trimonthly for patients 5 to 12 years (*n* = 136, 46.1%), 13 to 15 years (*n* = 78, 57.8%), 16 to 17 years (*n* = 66, 59.5%), 18 to 29 years (*n* = 124, 58.5%), and ≥ 30 years (*n* = 35, 47.9%), monthly testing for 1- to 4-year-olds (n = 78, 41.9%) and pregnant patients (n = 3, 60.0%), and twice a month for infants <1 year of age (*n* = 15, 39.5%). The frequency of testing compliance was over 50% in all countries in all groups except for 14–17 years old group and the 18–29 group in Mexico (Fig. 2a.4). For Argentina, in the <1 year old group the proportion of compliance was close to 90%. Conversely, in the 1–4 years old group (58%), the 13–17 years old group (62%) and the ≥30 years old (64%), the proportion of adherence was lower than for the other groups. Concerning Brazil, the highest adherence was found in the 1–4 years old group (85%) and in the 13–17 years old group (84%, while the lowest were obtained in the 5–12 years old (72%) and the ≥30 years old (69%) groups. Lastly, for Mexico, the proportions were between 67% (5–12 years old) and 75% (< 1 year old). (See Fig. 3.)

#### 3.5.3 Frequency of consultations: clinic recommendations and patient adherence

The frequency of clinical visits recommendations varied from weekly to twice a year for all age groups and pregnant women. For infants <1 year of age (*n* = 8, 42.1%) and pregnant patients (n = 7, 41.2%), the most common frequency of clinic visits recommended by each site was twice a month. >50% of the patients between 5 and 17 years old had trimonthly clinical visits. Around two-thirds of the adult patients had clinic visits twice a year or less often: 18–29 years old (*n* = 141, 66.2%) and patients ≥30 years old (*n* = 47, 67.1%).

### 3.6 Blood Phe concentration testing methodology

All 20 participating sites reported having a protocol in place on how to measure blood tests to assess Phe levels for PKU patients. Of these, 80.0% (*n* = 16) used Dry Blood Spots (DBS) on filter paper as the sampling method for measuring Phe, and the remaining 20.0% (n = 4) used EDTA test tubes. Regarding the method used to analyze Phe levels in these laboratories, >60% (*n* = 12, 63.2%) used the fluorometric method, 15.8% (*n* = 3) used the high-performance liquid chromatography (HPLC), and near 20% used other analysis method (n = 4, 21.1%). If considering only the distribution of the method to analyze Phe levels when DBS is the sampling method, most used the fluorometric method (*n* = 11, 68.8%) and tandem mass spectrometry (*n* = 2, 12.5%). Also, in three-quarters of the sites (*n* = 15), their institution or institution laboratory was responsible for analyzing Phe levels. For 15.0% (n = 3), the analysis was the responsibility of a third-party lab or private partner, and for 10.0% (n = 2) of the sites, a public laboratory did the analysis.

### 3.7 Impact of COVID-19 on PKU management

Clinicians were surveyed on the impact of COVID-19 on PKU management. All participating sites (*n* = 20) did believe that the frequency of visits to their clinic or frequency of exams was affected by COVID during the previous year (2020). More specifically, 55.0% of the sites (n = 11) considered that less than half of their patients were negatively impacted by COVID, 30.0% (*n* = 6) considered more than three-quarters of patients were negatively affected and 15.0% (n = 3) considered this percentage of negatively impacted patients to be between 51% and 75%. The most impacted areas were the frequency of visits/exams and treatment adherence.

## 4 Discussion

This study intended to characterize PKU patients and management practices in three reference countries in LatAm. The criteria for inclusion focused not only on NBS coverage -since countries like Cuba and Chile have excellent coverage and were not included, but also overall population size of the countries which are limited in these countries. In this regard, Colombia was not included since their NBS is more recent and therefore there is less experience with the management of PKU. Lastly, the countries of choice have both public and private health systems, which is of added value to the present analysis. From an initial pool of 40 selected reference regional sites in Argentina, Brazil, and Mexico, 20 sites responded to the survey, actively managing 1077 patients with PKU. This is relevant given that this reflects approximately a quarter of the estimated patient populations followed in those countries (2793 patients distributed in 32 clinics in Brazil, 1645 patients distributed in 12 clinics in Argentina and 124 patients distributed in 2 clinics in Mexico), as determined from internal unpublished market research data available from the study sponsor.

The results spanning across the surveyed countries show that guidelines recommending a lower blood Phe target of 120 μMol/L are widely implemented across age groups and for those pregnant or planning to become pregnant. Greater variability was found in the clinic-recommended upper threshold for target blood Phe concentration. For patients under the age of 4, all sites recommended an upper target of 360 μMol/L or less. Yet for patients aged 13 years and above, an increasing number of sites recommended an upper target above 360 μMol/L, approaching approximately 70% of sites for adult patients. This is a concern as recent research shows that an upper target goal of 360 μMol/L, results in a sustained reduction of Phe levels, leading to better clinical outcomes, as demonstrated by the reduction of inattention symptoms [19] in adults.

Results from the study characterizing pregnant or intending to become pregnant women were concerning. Survey results showed -although the number of patients in this condition is small- that 50.0% of pregnant patients were reported to have blood Phe levels above the recommended target. This is a concern since elevated blood Phe levels during pregnancy are not recommended as they have a teratogenic effect on the developing fetus and may lead to growth retardation, microcephaly, intellectual disability, and birth defects such as congenital heart defects [20]. Considering the significant percentage of pregnant women outside the recommended therapeutic target in this study, special attention is needed when dealing with women with PKU of childbearing age and during pregnancy, with consideration for including pharmacotherapy to improve metabolic control [7]. However, it must be stressed that these are probably among the first ladies benefiting from screening in the region, which does not fully justify the low awareness on the risks of high Phe levels during pregnancy. There was a lack of data on the number of women with PKU intending to become pregnant [21] and the current results shed some light on the subject. Previous research suggested that women with PKU that had planned their pregnancies were able to achieve more optimal blood Phe control within the recommended targets leading to better pregnancy outcomes [22]. However, having planned pregnancies can be more challenging [23,24]. Unwanted/unplanned pregnancy remains an issue in LatAm [25,26], and this is particularly important in PKU management. It is paramount to improve access to counseling, family planning, and patient education to ensure better adherence. In the US [15], more recent publications suggest lower blood Phe targets for pregnant/planning to be pregnant women to support improved fetal outcomes, which has implications on clinical management. Despite these more restrictive guidelines, the US faces similar challenges with a high proportion of unplanned pregnancies. The efforts to provide counseling and the implementation of a maternal PKU clinical and psychological support program are strategies employed for better adherence and other psychosocial effects in this specific group [27,28].

In patients as young as 5 years of age, there was a significant proportion that was non-adherent to the blood Phe targets as defined by their clinic, illustrating the challenges with following the Phe-restricted diet. This becomes progressively worse as patients age; by 16 years of age or older, >80% had an average blood Phe level above 360 μMol/L. This coincides with findings reported in other studies [15,[29], [30], [31]], demonstrating the need to define more specific approaches to improve adherence to the management of PKU in adolescent and adult patients. In Brazil [9], the lack of adherence to management recommendations has been identified as an issue that must be addressed. The treatment/follow-up discontinuation rate of 26% is comparable to results found in Japan [32]. Results on the proportion of patients over the target Phe range (roughly 50%) are similar to previous research [33], but is higher than in Europe (35%) and lower than in the US (67%) [15]. The same concern has been expressed by other countries in the region, namely Argentina. Adult patients had a lower frequency of visits since most are followed in pediatric clinics. However, as previously mentioned, the results for Argentina should be carefully interpreted in the context of less restrictive values of the upper limit, which indicate that the proportion of non-adherence in the country is much lower when considering an upper limit of 600 μMol/L. In other countries/regions, such as in Europe, adherence to management in adult patients has also been reported to be a concern, as guidelines recommend life-long dietary restrictions [7], though this has been reported not to be achievable in the majority of adult patients [29]. Moreover, there seems to be inadequate training for healthcare practitioners taking care of adult PKU patients, as shown in research in other countries [34], namely management of weight, overweight and protein requirements in adults.

The frequency of testing for each age group was concordant with the most frequently recommended occurrence of testing: trimonthly for patients >5 years, monthly for 1- to 4-year-olds and pregnant patients, and twice a month for infants <1 year of age. Furthermore, roughly 40% of the patients aged 30 years old or more had Phe blood tests done twice a year or less. Previous research from Brazil [9] suggests that testing frequency can be increased. And in comparison, to the ACMG guidelines, the frequency of testing in several of the age cohorts falls below these recommendations [3], with weekly testing for younger children (< 1 year old), bi-weekly to monthly for 1–12 years old and monthly testing for adolescents and adults with adequate dietary management. When compared to the European guidelines for pregnant women, which suggests weekly testing, the frequency of testing in LatAm is inadequately low [7]. Given the importance of monitoring blood Phe levels in PKU patients to achieve good clinical outcomes -in which dietary management is the cornerstone-, increased frequency of testing should be pursued in each country. However, Europe and the US face similar challenges regarding inadequate testing frequency for older age groups [35].

Concerning the number of clinic visits recommended by each participating site, these range from weekly to twice a year for all age groups and pregnant women. Twice a month was the most recommended frequency of clinic visits for infants <1 year of age and for pregnant patients. For the remaining age groups, the most recommended frequency of clinic visits was trimonthly with approximately 60% of sites recommending that adults <30 years old attend clinic twice a year. These recommendations are consistent with that recommend in the European guidelines [14] but results per country should be assessed as regional differences are evident between LatAm countries, namely Brazil and Argentina. Frequency of visits leads to early identification of dietary deficiencies and non-adherence and can promote better management of this condition, leading to better clinical outcomes (i.e. blood Phe concentration).

Regarding the impact of COVID-19 on PKU management, the physicians believe that the frequency of visits and exams were affected by COVID during the previous year. Regional evidence is scarce, but this impact was felt worldwide in chronic disease management [[36], [37], [38]]. However, some research in other regions [39] is aligned with current results, evidencing the detrimental impact in the screening and care given to patients. Conversely, the implementation of telehealth may have increased access to care for some patients that live far from the reference centers or have physical/economic limitations to travel. This is a positive outcome of the constraints posed by the pandemic and has the potential to streamline the communication between patients, their physicians and multidisciplinary healthcare team. This can also be performed beyond the use of formal communication strategies implemented at centers, as less formal and alternative communication tools (i.e., WhatsApp) have been shown to bring positive results in adherence and compliance [40].

### 4.1 Limitations

The selection of sites was based on a previous market research study carried out by the study sponsor for the identification of reference sites in the region. All viable sites were invited to avoid selection bias. The retrospectively collected observational aggregate patient-level data may be incomplete, poorly recorded, or absent. However, the recommended use of all available data sources and the presumably low number of cases followed at each site provides confidence in the findings. Moreover, there may be a memory bias, although clinicians were requested to access the site's database to increase accuracy. Conversely, the use of the aggregate data provides benefits in the confidentiality and data privacy, and in the interpretation and reporting of the data. However, this has an impact on the level of detail that can be drawn from the analysis of individual patient data. The fact that NBS occurred at different times in the countries under study (ARG: 2000; BRA: 2001; MEX: 2011), the differences in coverage in each country concerning medical foods and blood Phe testing, access to specialized care, national coverage of the sites, PKU management guidelines and even cultural issues, limit the direct comparison between countries in the region. Also important are the structural organization of each country's healthcare system, as well as noticeable regional differences within countries. Further research should be conducted on this matter to ensure a sound nationwide characterization. The COVID-19 pandemic negatively impacted people's lives, healthcare services, and patient care, as well as the results found in this study. However, since data was collected retrospectively, reported adherence shouldn't have been mischaracterized. The use of standardized values for the Phe range for the overall results limits country-level interpretation. The significant weight of Brazil in the study sample size may skew the overall results. Therefore, the authors decided to provide some country-level data to improve accuracy. The association between patient adherence to PKU management practices and staffing characteristics was also assessed. However, the results obtained were counterintuitive and hard to interpret. Therefore, the authors choose not to report them.

## 5 Conclusions

Global adherence to PKU practices in this regional study shows that it must improve in all ages, especially in adult and pregnant patients. Recent initiatives to increase NBS coverage seem to be improving outcomes, namely earlier diagnosis and treatment of PKU. This allows for prompt initiation of treatment avoiding the most severe consequences of PKU. Nevertheless, continued efforts should be put on patient and multidisciplinary teams or physician education -to improve knowledge and skills in clinical and dietary management-, availability of financial support for dietary options essential for PKU management, and staffing. Limited availability of therapeutic options also constitutes a concern, as well as to better understand the motivating factors and beliefs that influence dietary adherence in PKU. Regional differences are relevant, but asymmetries within each of the surveyed countries should be further explored in future publications. The difficulty in guaranteeing a nationwide coverage of reference centers is complex given the size of the countries surveyed. Enhancing access to specialized care -namely in adults- and increasing financial support can help to support increased adherence in the region. Results from this research help to characterize the epidemiological profile, to depict the level of support and resources needed- of the PKU population in three countries in Latin America- and to map the main health issues and challenges of PKU patients. It adds to the existing literature concerning the challenges for healthcare providers with managing these patients. This can support the interpretation of the health status of PKU patients and identify critical points that need quick solutions. The most important and obvious need is for the development of consistent guidelines across LatAm like the ones currently available in the US and EU.

## Declaration of Competing Interest

AC: The author reports no Conflict of Interests.

NS: The author has been engaged as a speaker and received fees and travel support from Biomarin.

MP: The author reports no Conflict of Interests.

MVA: The author received speaker fees and travel support from pharmaceutical companies.

EJ: The author declares that she was a former employee of BioMarin and is a BioMarin stock owner.

DRFV: Currently an employee of BioMarin.

DM: The author declares that she was a former BioMarin employee and a Biomarin stock owner from 2016 to 2021.

GCC: The author reports no Conflict of Interests.

HE: The author reports no Conflict of Interests.

KHN: The author received Travel support from Perkin Elmer.

MLA: The author reports no Conflict of Interests.

RBC: The author reports no Conflict of Interests.

TA: Received honoraria related to data collection.

IVDS: The author reports no Conflict of Interests.

## Data Availability

The data that underlie the results reported in this article (including text, tables, figures, and appendices) will be made available together with the research protocol and data dictionaries for non-commercial and academic purposes. After publication, additional supporting documents may be available for six months to two years. For more information, see BioMarin Data Sharing Web Page at https://www.biomarin.com/our-science/funding-and-support/publication-data-request/
